# RegExpBlasting (REB), a Regular Expression Blasting algorithm based on multiply aligned sequences

**DOI:** 10.1186/1471-2105-10-S6-S5

**Published:** 2009-06-16

**Authors:** Francesco Rubino, Marcella Attimonelli

**Affiliations:** 1 Department of Biochemistry and Molecular Biology "E. Quagliariello" – Bari, 70126, Italy

## Abstract

**Background:**

One of the most frequent uses of bioinformatics tools concerns functional characterization of a newly produced nucleotide sequence (a query sequence) by applying Blast or FASTA against a set of sequences (the subject sequences).

However, in some specific contexts, it is useful to compare the query sequence against a cluster such as a MultiAlignment (MA). We present here the RegExpBlasting (REB) algorithm, which compares an unclassified sequence with a dataset of patterns defined by application of Regular Expression rules to a given-as-input MA datasets.

The REB algorithm workflow consists in

i. the definition of a dataset of multialignments

ii. the association of each MA to a pattern, defined by application of regular expression rules;

iii. automatic characterization of a submitted biosequence according to the function of the sequences described by the pattern best matching the query sequence.

**Results:**

An application of this algorithm is used in the "characterize your sequence" tool available in the PPNEMA resource. PPNEMA is a resource of Ribosomal Cistron sequences from various species, grouped according to nematode genera. It allows the retrieval of plant nematode multialigned sequences or the classification of new nematode rDNA sequences by applying REB. The same algorithm also supports automatic updating of the PPNEMA database. The present paper gives examples of the use of REB within PPNEMA.

**Conclusion:**

The use of REB in PPNEMA updating, the PPNEMA "characterize your sequence" option clearly demonstrates the power of the method. Using REB can also rapidly solve any other bioinformatics problem, where the addition of a new sequence to a pre-existing cluster is required. The statistical tests carried out here show the powerful flexibility of the method.

## Background

One of the most frequent needs a Bioinformatics end-user has is to characterize a newly produced sequence. In addition, the design and implementation of specialised bio-databases is a very frequent research activity, as demonstrated simply by accessing the NAR Database Issue compilation [1], but the main problem is keeping bio-databases updated in time. Both these needs – new sequence characterization and bio-database updating, are not always easy to satisfy fully. Indeed, characterization of "newly produced sequences" is usually performed by applying database similarity searching methods, such as BLAST [2] and FASTA [3], which are based on heuristic algorithms. The results, besides being approximate, then require further semi-automatic management in order to characterize the new sequences clearly, as regards their taxonomic and functional location. A great advantage in the "new sequence" classification procedure may be achieved by comparing new sequences with a set of already classified and grouped multialigned sequences, the grouping being based on already assigned functions and species. Here we define this approach as the One-Sequence *vs *a Dataset of more(n) Multi-Alignments (OS/DnMA). The rationale of the OS/DnMA approach has been implemented in other programs such as HMMalign within PFAM [4] or HoSeqI and MultiHoSeqI [5], but both these approaches are protein oriented. As far as the nucleotide sequences, the already available programs based on the OS/DnMA approach are the Classifier available through the Ribosomal Database Project Portal [6] and the ChiSeqI [5] both designed to characterize 16srRNAs sequences only. This paper describes an algorithm that was designed to respond to requirements for implementing a tool for characterization of newly sequenced plant parasitic nematode rRNA genes. Indeed, after the PPNEMA database had been developed [7,8], we had not only to demonstrate the advantage of having the PPNEMA database available for phytoparasite nematologists, but also to implement a PPNEMA updating procedure as efficient and automatic as possible. PPNEMA, a **Plant-Parasitic Nematode **bioinformatics resource, offers the scientific community a pre-processed archive of plant parasitic nematode sequences useful for nematologists: it is a tool for retrieving plant nematode multialigned sequences for phylogenetic studies or identifying a nematode by comparing its rDNA sequence with the PPNEMA available, genus specific MultiAlignments (MA). Classification of a new sequence by applying BLAST to all the sequences available through PPNEMA should have required an *ab-initio *procedure not considering already available multialigned groups. RegExpBlasting (REB), the algorithm proposed here, which compares a new sequence against a dataset of patterns derived from the application of Regular Expression coding to multialigned sequences, successfully solves this problem. In addition, as regards PPNEMA database updating, starting from the new rDNA cystronic phytoparasite nematode sequences extracted through the Entrez retrieval system [9], available at NCBI and not yet stored in PPNEMA, we implemented a protocol, based on REB, which allows each new sequence to be assigned to the best matching PPNEMA MA. RegExpBlasting is not implemented only for its specific application within PPNEMA but, as reported below, it can be more generically applied to any set of both nucleotide and amino-acidic multi-alignments with the aim to characterize a new sequence. However we have successfully tested its usage in the application to nucleotide sequences.

## Results

### Characterization of a new phytoparasite sequence

**Figure 1:**
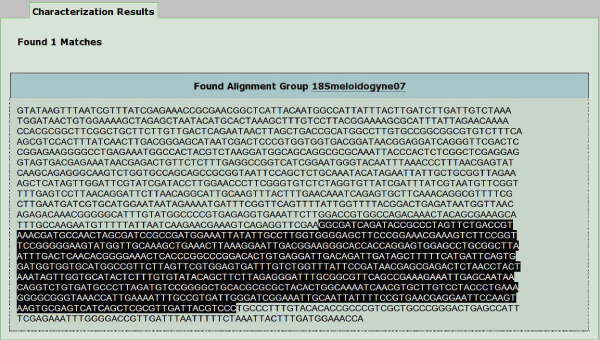
**Characterization of a new sequence**. A new nematode sequence is submitted to "characterize your sequence" option available through PPNEMA search page. Analysis shows a perfect match between the submitted sequence and part of 18s cistron element meloidogyne group 07 multi-alignment available in PPNEMA.

**Figure 2:**
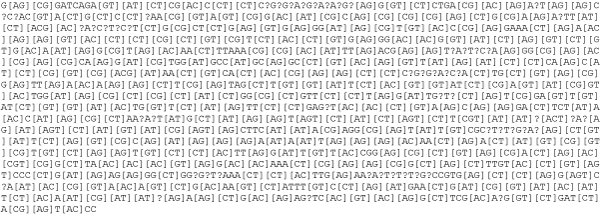
**Regular expression describing the MA of the Meloidogyne genus 18s group 07 available in PPNEMA**.

When phytoparasite nematologists produce a new sequence, they need to classify it as regards both function and species. The application of REB through the "characterize your sequence" option available in PPNEMA is of great help in this case. The algorithm searches for a perfect match between the query sequence and PPNEMA multi-alignments. An example is shown in Figure 1, in which the matching region is highlighted; Figure 2 reports the RegExp pattern describing the matching MA.

### Use of RegExpBlasting for the PPNEMA database updating

**Figure 3:**
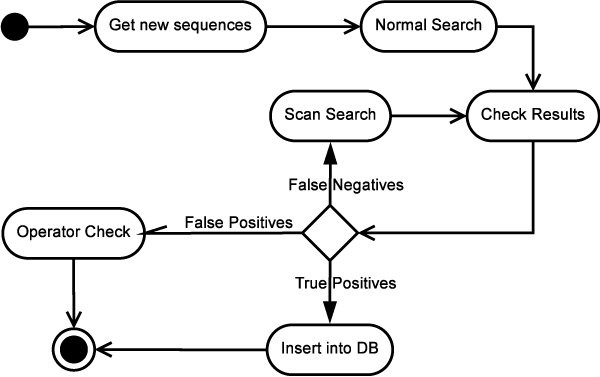
**Work-flow of PPNEMA updating procedure**. Work flow starts with extraction of new phytoparasite nematode sequences available in GenBank. Sequences are submitted to REB in "Normal Search" version. Results are checked and sorted into TP, FP and FN. TP are annotated in PPNEMA, FN are submitted to Scan-REB and FP are analysed by an operator.

**Figure 4:**
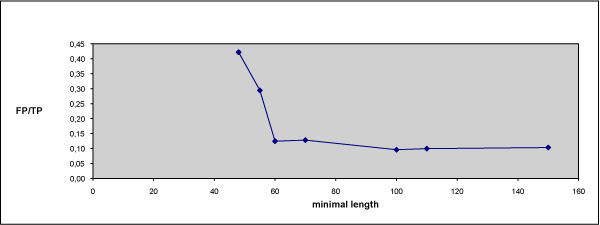
**False positive (FP)/True Positive (TP) versus Minimal lengths in the RegExpBlasting application to PPNEMA updating**. With increasing minimal length, FP decrease, although variations are unimportant for minimal length greater than 60; minimal length was therefore fixed to 60.

As already emphasized in the Background, in bio-database management, the main problem is to keep the databases updated in the time. We present here a procedure, based on REB, which allows the PPNEMA database to be updated in an almost completely automatic process. Figure 3 reports the workflow of the entire process. The protocol starts by searching, through Entrez implemented at NCBI the nematode sequences whose entry date is later than the most recent PPNEMA updating. The sequences are extracted by grouping them according to genus. An example of a query is "Anguina [ORGN] AND (18S OR 28S OR 5.8S OR ITS1 OR ITS2) AND 2007/01/01:2008/01/01 [PUBLICATION DATE]". The resulting entries refer to the nucleotide sequences from Genera *Anguina*, coding for one or more elements of the RNA cistron and annotated after January 1 2007 and before January 1 2008. They are extracted, and a file containing their nucleotide sequences in FASTA format is submitted to "Normal Search", which is based on RegExpBlasting of each of the selected sequences against the PPNEMA MA dataset. Each new sequence is associated with a pool of matching patterns that we call "positives"; any unclassified sequences or negative results are marked as True Negatives (TN), i.e. new group-defining sequences, and False Negatives (FN). In the first application of the protocol described here, we obviously carried out many checks in the various steps, in order to verify the efficiency of the protocol as we have designed it, and to optimize it. The positives are submitted to an automatic annotator check, which establishes if the results are true positives (TP) or false positives (FP). TP entries, i.e. entries matching MA of the same genera and related to various elements of the same cistron, are annotated in PPNEMA. The FP are submitted to an Operator Check and distinguished into FP due to annotation errors, e.g. erroneous species attribution, or to short conserved among-genera sequences. In the latter case, the same sequence matches several genera; thus, to solve the problem, RegExpBlasting can modify the minimal length parameter (see Methods). By increasing the minimal length, the probability of random matches decreases, although some significant but short matches may be lost. Figure 4 shows that the minimal length 60 is a good compromise, yielding as many TP as possible and minimizing the number of FN. This implied exclusion from analysis of dataset patterns whose minimal length was less than 60. This value is applicable to the 884 phytoparasite sequences extracted. The algorithm allows minimal length to be changed dynamically, depending on the dataset in question. Once the minimal length is established, the protocol is tested by submitting the FN to the Scan-RegExpBlasting procedure, in which the parameters used are window length **w**, scanning step **s **and minimal length **minl**. Once fixed **minl **to 60, the results which show a cut-off (**cf**) value higher than 0.70 are selected (see Methods).

We tested the procedure using window values of 20, 30 and 40, and tested for each window steps 10, 15 and 20.

**Figure 5:**
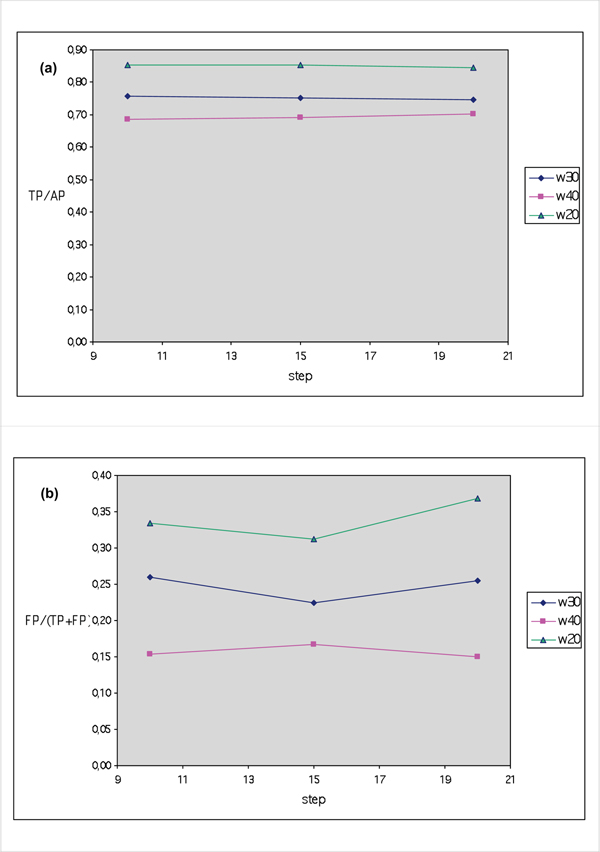
**(a) Selectivity (TP/(TP+FN)) trend and (b) False positives (FP)/(True Positives (TP) + False Positives (FP)) versus step values at various window lengths in the RegExpBlasting application to PPNEMA updating**. Window length 20 clearly shows best performance, independent of step value.

**Figure 6:**
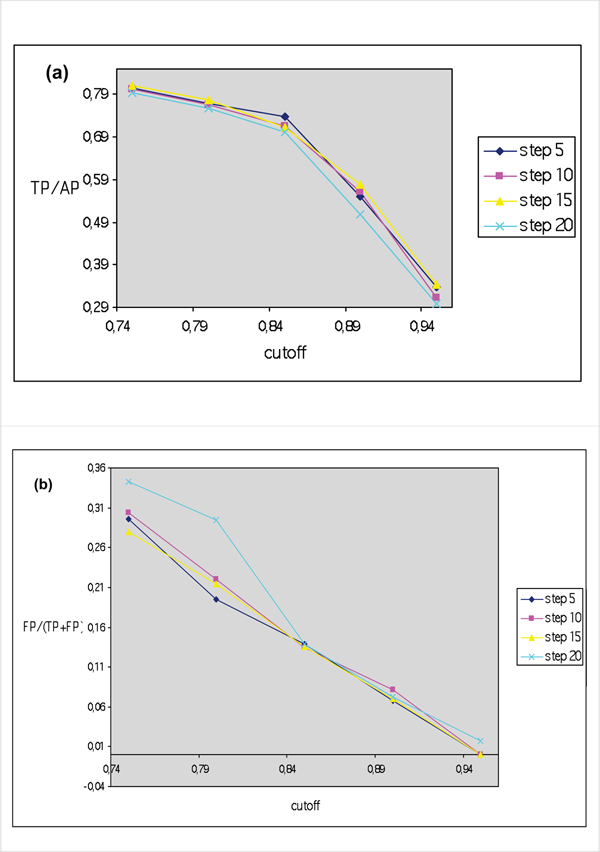
**(a) Selectivity (TP/(TP+FN)) trend and (b) False positive (FP)/(True Positives (TP) + False Positives (FP)) versus cut-off values at various step values, with minl fixed at 60 and window length at 20**. Cut-off value of 0,85 produces the best results regards selectivity. Once again, step changes do not produce evident variations in number of positive results.

Figure 5a reports the sensitivity of the method (TP/AP) versus step s for three different window values. Clearly, within the same w value, step s has no influence. The shorter the window, the higher the sensitivity, but also the higher the number of FP (Figure 5b), although there is a minimal but still detectable variation when the step changes. Thus, once minimal length (minl = 60) and window length (w = 20) had been fixed, we observed the effect of various cut-off values and steps on selectivity and sensitivity. Figure 6a shows that i) step changes have no influence on the number of positives results, but the cut-off value plays a determinant role and, when the cf is higher than 0.85, the number of TP is drastically reduced. Thus, although the number of FP could be reduced by increasing the cf value, it is more convenient to fix it at 0.70 and work with step values not higher than 15.

However in the end, we fixed the Scanning procedure parameters as follows: minl = 60, w = 20, s = 5 and cf = 0.85. Table 1 lists the results of the entire updating procedure. Additional file 1 gives the list of 184 FN after Normal Search. Additional file 2 reports the table summarising the results of the Scan-RegExpBlasting procedure applied to the abovementioned FN.

**Table 1:** Quantitative report of the PPNEMA updating procedure

Number of selected GenBank entries	884
Number of PPNEMA Groups considered	245
Number of TP in Normal Search	1294
Number of FN in Normal Search	184
Number of TP after Scan-RegExpBlasting	155
Number of True Negative (TN)	29

Data refer to application of entire RegExpBlasting procedure, as described in Figure 2, with following fixed parameters: **minl **= 60, **w **= 20, **step **= 5 and **cut-off **= 0.85.

## Discussion

It has been emphasized that REB is neither an exact matching method nor a heuristic one but, thanks to careful selection of minimal length and cut-off values, as well as scanning parameters (w and s), results mainly fitting true results can be obtained. What are the limitations? One of the most difficult problems that can be avoided concerns the quality of the MA: non-optimised MA can give rise to increased numbers of False results, both positive and negative. In addition, a poor-quality MA may introduce a great number of gaps, implying reduction of minimal length, elimination of the cluster to be analysed from the dataset, and hence reduction of informative data necessary to allow positives to grow. The solution is to lower the minl value but this, as shown in Figure 4 causes an increase in false positives. Thus, in phase I it is worthwhile excluding MA which may reduce the minimal length or, more generally, the growth of false results. Lastly, as regards MA, a great number of gaps inside them may lead to an increase in the CPU time of the run.

## Conclusion

The results here presented and the description of the algorithm clearly show that REB is a tool with a wide range of applicability. Besides the example shown here, we hypothesize that it may be used to definite advantage in Barcoding [10] procedures in order to identify the best matching group of sequences for a new sequence. Moreover we plan to introduce this code in the HmtDB resource [10-12] to contribute in the classification of human mitochondrial (mt) haplogroups when a partial sequence of the human mt genome is produced. Finally it will be tested in the case of sequences where alternative splicing is present, using the Scan option of the REB software. We have not yet verified this hypothesis, but we are confident that it would be successful.

## Methods

Similarity searching algorithms are usually classified as exact or heuristic methods.

Both methods compare an anonymous sequence against a dataset of sequences available in bio-databases. Exact methods search for the perfect match between an anonymous sequence and dataset sequences. Heuristic methods, i.e., FASTA and Blast, compare sequences by searching for matching substrings.

However, biological sequences, due to their intrinsic features, the presence of SNPs, insertions and deletions, cannot be managed well with exact methods. And although heuristic methods provide many results, many of them may be approximate or false, so that end-users have to check all results, if possible discriminate false from true positives, and then select the result that gives the best match with the queried sequence from among the true positives.

RegExpBlasting is a valid alternative to exact matching methods because, if positive results are obtained, they will be formed by a set of sequences grouped into an MA described by a pattern written through regular expression rules. Thus, RegExpBlasting is based on the widely used Regular Expression syntax, by which better than with the consensus definition, it is possible to describe the degeneration of a biological pattern defining a specific function.

The starting point in the generation of the regular expression within biosequence management is a sequence MA.

The algorithm is made of two phases: one which generates regular expressions starting from the MA, and the other based on the search of the MA whose regular expression pattern best describes the sequence to be characterized. The algorithm is organized in two phases. RegExpBlasting phase I produces the RegExp pattern describing each of the considered multialignments by extracting the regular expression which represents all the variations of the group of sequences available through their MA. RegExpBlasting phase II allows the classification of a new sequence by comparing it with each of the patterns defined in phase I and reports as output the matching MA to which the new sequence can easily be added.

### Phase I – Regular expression generation

Regular expressions are tools used to represent every possible character variation in a sequence alignment, and are much more powerful than the classic consensus sequences which leave out a great quantity of information about sites such as possible SNPs, insertions and deletions. RegExpBlasting is an algorithm which can characterize a new sequence simply by comparing it with the regular expressed patterns defining the queried MA. Starting from each of the MA to be browsed, the nucleotide or aminoacid composition of each variant MA site is calculated, inclusive of gaps: this datum allows the site to be translated into regular expression annotation. The rule is: the entire site composition is associated with a character class, within square brackets; if gaps occur in the site, a question mark is added to the right of the character class. If the site is invariant, it is represented by the nucleotide followed by "?" if gaps occur in the site in question. If the MA is made of non-conserved sequences, displaying large gapped regions due to indel events among the MA composing sequences, the regular expression in the indel regions allows any character, i.e., a wild card. For this whole region, RegExpBlasting implements two different options: a) a number of wild cards less than or equal to the length of the indel region or b) a number of wild cards of any length. From a biological viewpoint, this implies that the indel regions, being so variant, have no importance in the function of the multialigned sequences in question.

In this way, each MA is translated into a Regular Expression, representing every sequence composing that MA and thus generating the RegExp datasets against which "blasting" of the query sequence can be applied. In order to use RegExpBlasting to best effect, it is important for end-users to be aware of several aspects which lie at the basis of pattern generation. These are minimal length and the number of gapped sites. Minimal length **minl **is the shortest sequence, which can produce a match, and is calculated as the difference between alignment length and number of gapped sites. It is closely correlated to the number of false positives, as will be demonstrated in the description of phase II. The number of gapped sites strongly influences the processing time of the algorithm: the higher the number of gapped sites, the longer the processing time.

### Phase II – Blasting of a query sequence against the RegExp dataset

In this phase, RegExpBlasting compares the query sequence against the RegExp dataset, and the resulting output is the MA associated with the best matching pattern. When no positive results are obtained, a further analysis can be performed through the Scan-RegExpBlasting procedure. This procedure scans each RegExp dataset-composing pattern, using a window **w **characters long and a step **s **characters long, thus generating **n **sub-patterns which can then be analyzed. Both **w **and **s **are parameters which can be modified by end-users. Thus, once cut-off **cf **(i.e., the minimum number of matching windows related to the total number of scanned windows which must be detected in a pattern in order to define it as a positive result) has been fixed, the algorithm selects the positive matches if the number of matched sub-patterns related to the total number of considered sub-patterns is equal or greater than the fixed cut-off. In the first application, several tests were carried out by changing the minimal length, window and step length; in order to evaluate parameter values that optimize the performance of the algorithm and define general criteria for end-users.

### Implementation

The algorithm is written in python [13] but a Web interface will soon be implemented allowing end-users to generate regular expressions and RegExp datasets starting from the MA they submit. The Web system will also allow the characterization of new sequences against end-users defined datasets. Obviously, default parameters will be fixed, but end-users will be allowed to modify them. At present the software can be downloaded and locally used through the tool section implemented within the PPNEMA resource [14].

## List of abbreviations used

MA: Multiple Alignments; REB: Regular Expression Blasting

## Competing interests

The authors declare that they have no competing interests.

## Authors' contributions

FR has designed and implemented the algorithm with the advise of MA. MA has interpreted the results and wrote the manuscript.

## Supplementary Material

Additional file 1 **List of False Negative GenBank entries**. List shows GenBank ID and species name of entries extracted through Entrez, submitted to PPNEMA RegExpBlasting "Normal Search" modality (see figure 2 in text) and resulting as False Negatives (FN). Click here for file

Additional file 2 **Report of the Scan-RegExpBlasting procedure applied to FN listed in Aditional file **1. Excel sheet lists species names, GenBank ID, matching PPNEMA group ID, number of matching windows, total number of windows, and percentage of matching windows. Click here for file
